# Maternal cadmium exposure in the mouse leads to increased heart weight at birth and programs susceptibility to hypertension in adulthood

**DOI:** 10.1038/s41598-019-49807-5

**Published:** 2019-09-19

**Authors:** Kathleen M. Hudson, Scott M. Belcher, Michael Cowley

**Affiliations:** 1 Center for Human Health and the Environment, and Department of Biological Sciences, North Carolina State University, Raleigh, NC 27695 USA; 2 W. M. Keck Center for Behavioral Biology, North Carolina State University, Raleigh, NC 27695 USA

**Keywords:** Hypertension, Disease model, Gene expression, RNA sequencing

## Abstract

Cadmium (Cd) is a toxic heavy metal ubiquitous in the environment. Maternal exposure to Cd is associated with fetal growth restriction, trace element deficiencies, and congenital malformations. Cd exposure during adulthood is associated with cardiovascular disease (CVD); however, the effects of maternal Cd exposure on offspring cardiovascular development and disease are not well-understood. Utilizing a mouse model of maternal Cd exposure, we show that offspring born to Cd-exposed mothers have increased heart weights at birth and susceptibility to hypertension during adulthood. Despite inefficient maternal-fetal transfer of Cd, maternal Cd alters fetal levels of essential trace elements including a deficiency in iron, which is required for cardiovascular system development, oxygen homeostasis, and cellular metabolism. RNA-seq on newborn hearts identifies differentially expressed genes associated with maternal Cd exposure that are enriched for functions in CVD, hypertension, enlarged hearts, cellular energy, and hypoxic stress. We propose that a maternal Cd exposure-induced iron deficiency leads to altered cellular metabolic pathways and hypoxic conditions during fetal development; this stress may contribute to increased heart weight at birth and the programming of susceptibility to hypertension in adulthood. These studies will give insights into potential mechanisms through which maternal Cd exposure impacts cardiovascular development and disease.

## Introduction

The environment during development can impact health in later life, a concept called developmental programming^1^. Maternal exposure to heavy metals^2^, endocrine disruptors^3^, and over- or under-nutrition^4^ during gestation have all been shown to negatively affect the development and future health of offspring, even after cessation of exposure.

Cd is a toxic heavy metal found throughout the environment and is designated as one of the ten chemicals of major public health concern by the World Health Organization (WHO)^5,6^. Cd is naturally found at relatively low levels in the soil (0.1–0.5 parts per million (ppm))^7^, but higher levels can be found in clustered geographical locations due to natural sources or pollution^8,9^. Exposure to Cd has dramatically increased over the last century due to human activities such as fossil fuel combustion, metal mining and refining, tobacco smoking, and municipal waste incineration^10^. Humans are exposed to Cd through inhalation or ingestion. Cd inhalation occurs predominantly through smoking cigarettes, but can also occur through inhaling contaminated dust or through occupational exposure. Cd ingestion is the main source of exposure among non-smokers and occurs through the consumption of contaminated water or food^8,9^, particularly crop plants that absorb Cd from the soil. Produce that is recommended as part of a healthy diet especially during pregnancy, such as whole grains and leafy greens, is often the primary source of dietary Cd^11^. Cd is not required by any processes in the body and is poorly excreted, resulting in long-term storage in several target organs^7^. Females tend to absorb and accumulate more Cd in their bodies than males with Cd absorption being the highest during pregnancy, likely due to increased requirements for iron^12^.

Chronic exposure to Cd during adulthood has been shown to negatively impact cardiac health^13–15^. Several epidemiological studies have demonstrated an association between blood and urinary Cd levels and cardiovascular disease (CVD; e.g., heart disease and stroke), which is the leading cause of death and disability in the United States (US)^15,16^. Cd exposure increases oxidative stress^2^, a critical event in the pathogenesis of hypertension^17,18^. Uncontrolled hypertension and smoking are two of the three risk factors for CVD^19^.

While the association between Cd exposure during adulthood and CVD has been well-demonstrated in human and animal studies, only a few studies have examined the impacts of early-life Cd exposure on cardiac development and the programming of long-term CVD. Maternal exposure to Cd has been associated with fetal congenital heart defects in humans^20^ and altered heart morphology and endothelial function during adulthood in rats^2^. We recently showed in a human cohort that higher maternal blood Cd levels during pregnancy are associated with changes to cord blood DNA methylation at genes involved in cardiometabolic functions^21^, suggesting that maternal Cd exposure could affect their regulation and potentially influence cardiac development and health. However, it has not been established in humans or rodents whether these molecular and physiological changes are associated with impaired cardiac function or biomarkers of CVD.

**Figure 1:**
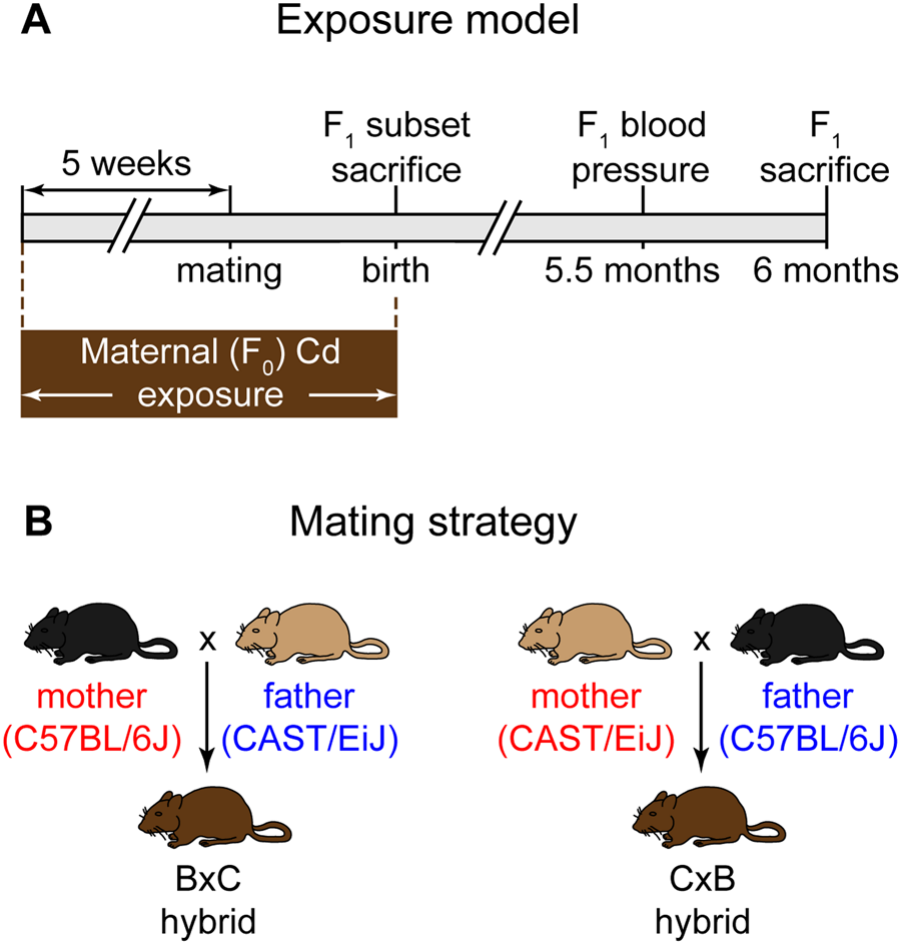
Maternal Cd exposure model and mating strategy to produce maternally-exposed hybrid F_1_ offspring. (**A**) Exposure model. (**B**) Mating strategy. B = C57BL/6J, C = CAST/EiJ. F_1_ hybrids are designated as maternal × paternal strain.

To address these outstanding questions, we have established a mouse model of maternal Cd exposure (Fig. 1) using a low, environmentally relevant Cd dose and a high dose of Cd that has been used in previous rodent studies^22–24^. We generated F_1_ mice from matings between two genetically divergent inbred strains to study allele-specific epigenetic and transcriptional responses to maternal Cd exposure. The results of these allele-specific analyses will be presented elsewhere. In the current study, we use this mouse model to show that maternal Cd exposure causes increased heart weight in the offspring at birth and that maternal Cd exposure alone is sufficient to program susceptibility to hypertension in adulthood. To inform on molecular mechanisms that may contribute to the observed phenotypes, we identify altered gene expression pathways and essential trace element levels at birth caused by maternal Cd exposure.

## Results

### Maternal Cd exposure results in decreased body weight and increased heart weight at birth

The exposure model and mating strategy are outlined in Fig. 1. One of three doses of Cd was given to F_0_ females through their drinking water: 0 ppm (control), 1 ppm (low, environmentally relevant dose), and 50 ppm (high dose previously used in other rodent studies). F_1_ mice were sacrificed at birth or aged to 6 months. For ease of annotation, the generation of the mice and the Cd dose to which they were exposed will be herein referred to as: F_(generation)_^(Cd dose)^. For example, F_0_^1ppm^ refers to mothers exposed to 1 ppm Cd and F_1_^50ppm^ refers to offspring maternally exposed to 50 ppm Cd. A hybrid mating scheme between two genetically divergent inbred strains of mouse, C57BL/6J (B) and CAST/EiJ (C), was employed to facilitate a separate study of allele-specific DNA methylation and gene expression changes in response to maternal Cd exposure, the results of which will be presented elsewhere. F_1_ mice will be herein referred to as B × C (B mother × C father) or C × B (C mother × B father).

Maternal Cd exposure did not influence litter size or sex ratio of the offspring (Supplementary Fig. 1; litter size: F_2,49_ = 1.250, p = 0.295 (B × C) and F_2,26_ = 0.377, p = 0.690 (C × B), one-way analysis of variance (ANOVA) comparing the three Cd doses; sex ratio: p = 0.943 (F_1_^0ppm^), p = 0.115 (F_1_^1ppm^), p = 0.633 (F_1_^50ppm^), chi-square test based on expected 1:1 male:female ratio). However, 50 ppm Cd was found to significantly increase the number of problematic deliveries for C mothers (5 problematic deliveries out of 17 total in C F_0_^0ppm^, 9 problematic deliveries out of 16 total in C F_0_^50ppm^ mothers; p = 0.019, chi-square test comparing to the number of problematic births of F_0_^0ppm^ mothers; Supplementary Table 1).

**Figure 2:**
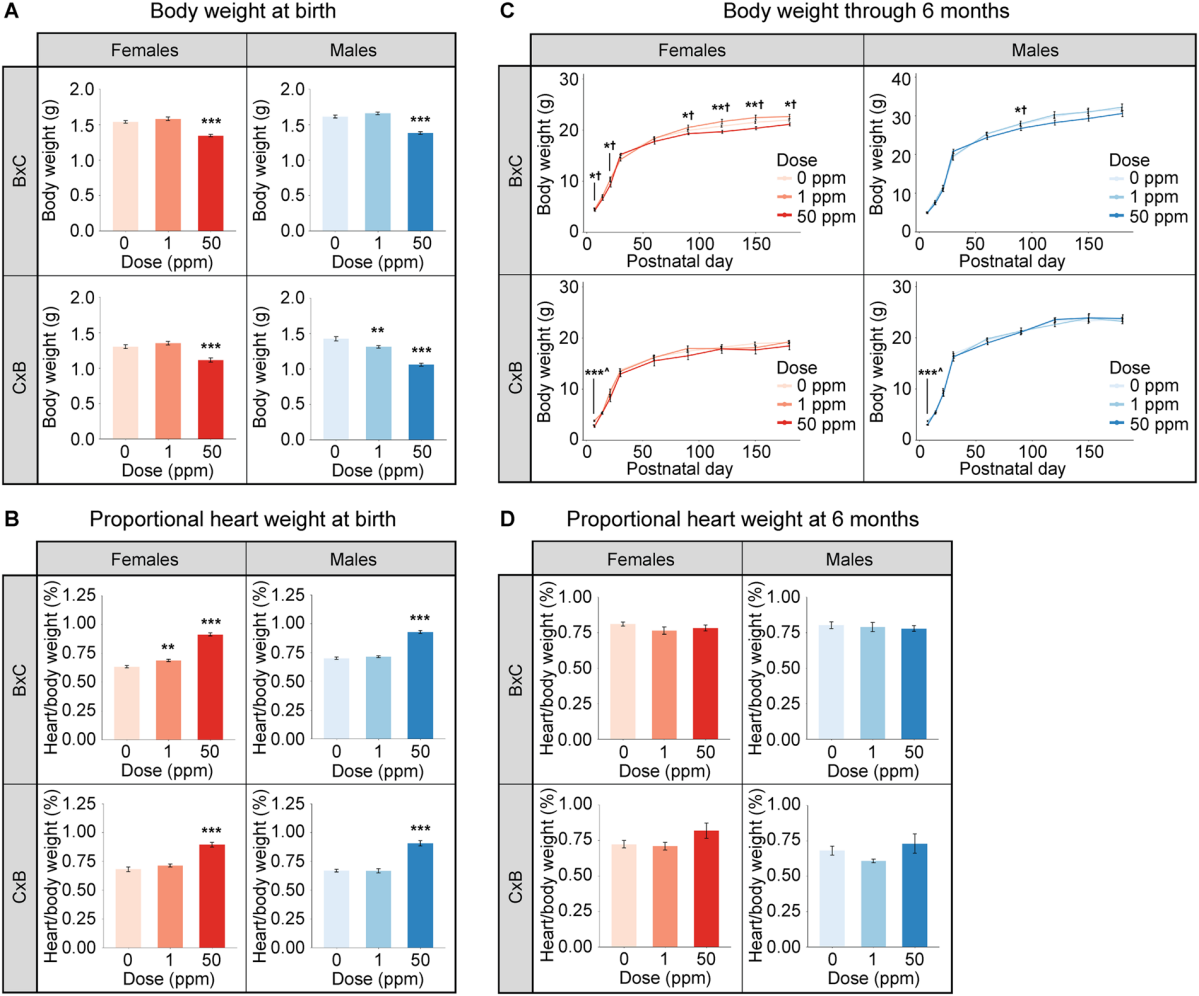
The effects of maternal Cd exposure on body weight and normalized heart weight at birth and adulthood. (**A**) Body weight at birth. (**B**) Proportional heart weight at birth. (**C**) Body weight through 6 months. ^ = 0 ppm vs 1 ppm, † = 0 ppm vs 50 ppm. (**D**) Proportional heart weight at 6 months. *p < 0.05, **p < 0.01, ***p < 0.001 (one-way ANOVA with post-hoc Dunnett’s test comparing 1 ppm and 50 ppm to 0 ppm).

The body weights of male and female F_1_^50ppm^ mice from both crosses were significantly reduced at birth compared to control animals (Fig. 2A; see also Supplementary Table 2), consistent with previous studies in mice using the same dose^25^. F_1_^1ppm^ C × B males also showed a significant reduction in body weight at birth (Fig. 2A and Supplementary Table 2).

Mice presented with increased proportional heart weight at birth due to maternal Cd exposure. This increase was significant for F_1_^50ppm^ mice compared to controls in both sexes and crosses. F_1_^1ppm^ B × C females also demonstrated a significant increase in proportional heart weight (Fig. 2B and Supplementary Table 2). Despite the reduction in overall body weight among the F_1_^50ppm^ mice, hearts in these animals were still significantly increased in raw weight compared to controls, except for C × B males (Supplementary Fig. 2A).

The body weight differences between control and exposed F_1_ mice observed at birth persisted beyond 7 days of age through 6 months of age only for F_1_^50ppm^ B × C females, with the exception of a significant decrease in body weight seen in F_1_^50ppm^ B × C males at 90 days of age (Fig. 2C). At 6 months of age, there were no significant differences in heart weight in terms of raw weight or relative to body weight (Fig. 2D and Supplementary Table 2), with the exception of a significant reduction in raw heart weight in F_1_^50ppm^ B × C females (Supplementary Fig. 2B).

### B × C females are susceptible to hypertension and altered blood pressure parameters during adulthood as a result of maternal Cd exposure

**Figure 3:**
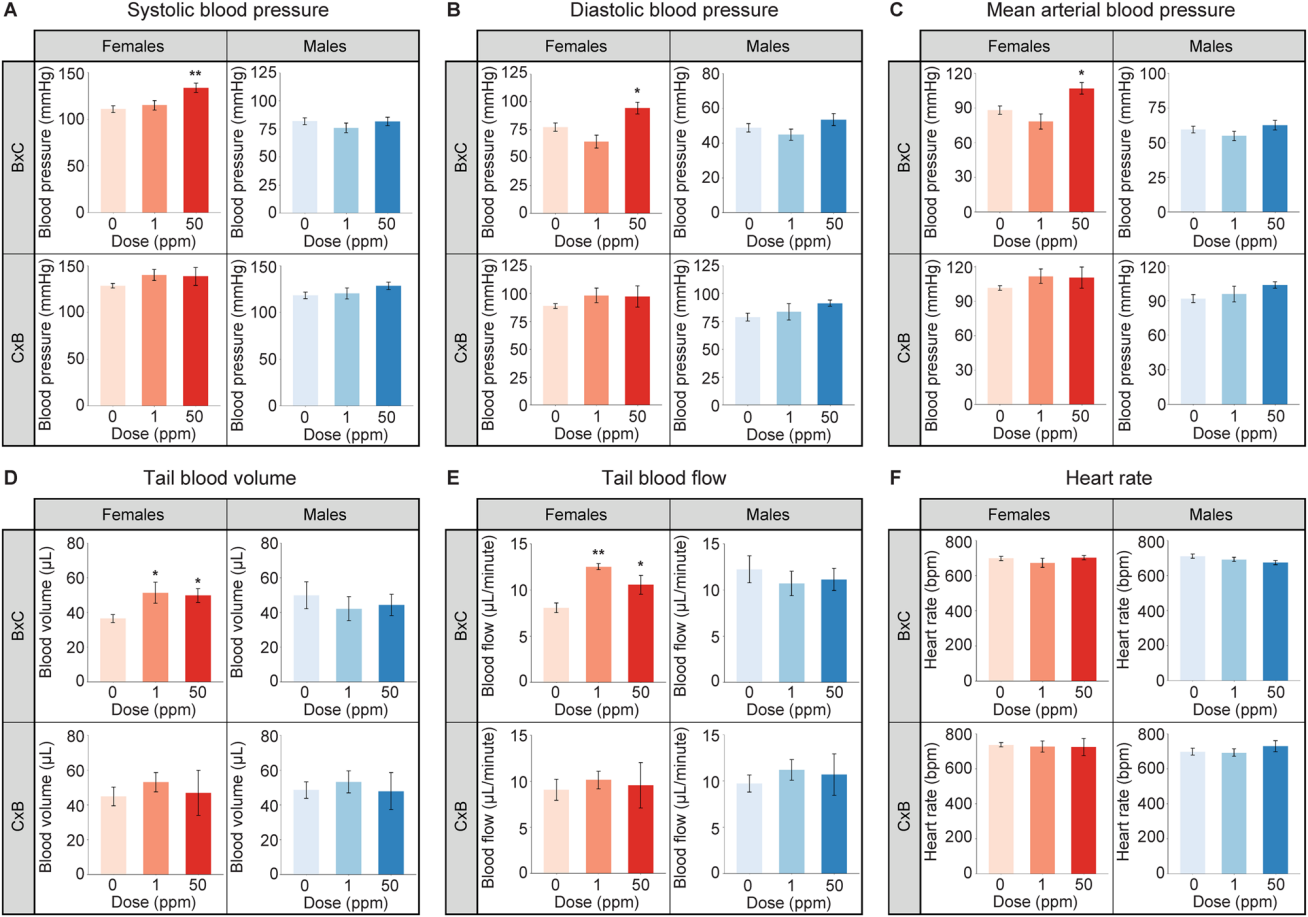
Blood pressure parameters in F_1_ animals at 5.5 months of age following maternal Cd exposure. (**A**) Systolic blood pressure. (**B**) Diastolic blood pressure. (**C**) Mean arterial blood pressure. (**D**) Tail blood volume. (**E**) Tail blood flow. (**F**) Heart rate. *p < 0.05, **p < 0.01 (one-way ANOVA with post-hoc Dunnett’s test comparing 1 ppm and 50 ppm to 0 ppm).

Due to the increased weight of the heart seen in the maternally-exposed F_1_ mice at birth and the previously reported association between adult exposure to Cd and hypertension in humans, blood pressure parameters were measured in 5.5-month-old F_1_ mice to test whether hypertension in adulthood can be programmed by maternal Cd exposure alone. F_1_^50ppm^ B × C females demonstrated significantly increased systolic, diastolic, and mean arterial blood pressures, consistent with a hypertensive phenotype (Fig. 3A–C and Supplementary Table 2). F_1_^50ppm^ B × C males, C × B females, and C × B males displayed increased systolic, diastolic, and mean arterial blood pressures, although these changes were not statistically significant (Fig. 3A–C and Supplementary Table 2). Both F_1_^1ppm^ and F_1_^50ppm^ B × C females showed a significant increase in tail blood volume and tail blood flow (Fig. 3D,E and Supplementary Table 2). There were no significant differences in heart rates between any of the groups (Fig. 3F and Supplementary Table 2).

### Circulating reactive oxygen species (ROS) levels are not elevated in maternally-exposed B × C females at 6 months of age

**Figure 4:**
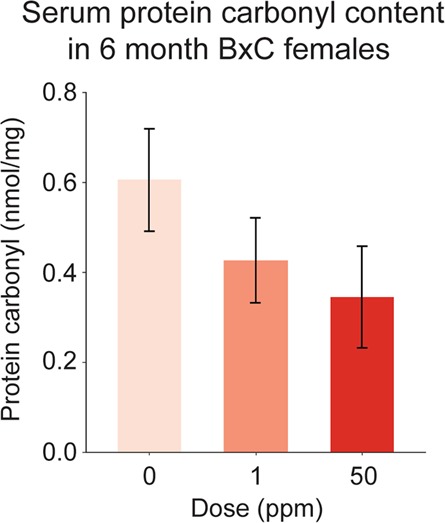
Protein carbonyl quantification in serum of 6-month-old B × C females.

We next sought to understand the molecular basis of the hypertensive phenotype in F_1_ B × C females to inform on the mechanisms through which maternal Cd exposure can program hypertension. ROS have been suggested to play a role in the toxicity of Cd exposure during adulthood^26^. In addition, elevated ROS levels in circulation and relevant tissues contribute to hypertension^27^. To determine if the hypertension seen in maternally-exposed B × C females was associated with elevated ROS levels in circulation, ROS were quantified at 6 months of age by measuring serum levels of protein carbonyl, a stable marker of ROS^28^. No significant differences in serum protein carbonyl content were observed between exposure groups (F_2,18_ = 1.488, p = 0.252, one-way ANOVA; Fig. 4), suggesting that circulating ROS levels were not elevated at 6 months of age.

### Essential trace elements associated with hypertension are not altered in maternally-exposed B × C females at 6 months of age

Maternal exposure to Cd has been shown to affect trace element levels in various tissues in the offspring at birth^29^. To determine if the hypertension observed in adult maternally-exposed B × C females was associated with abnormal homeostasis of essential trace elements known or suspected to contribute to blood pressure^30–34^, levels of calcium (Ca), iron (Fe), potassium (K), magnesium (Mg), sodium (Na), copper (Cu), selenium (Se), and zinc (Zn) were quantified in 6-month-old B × C female blood and liver tissue (Tables 1 and 2). Blood and liver tissue were chosen for analyses as the elements in question are detectable in circulation or stored in the liver. No significant differences in any elements were seen at 6 months of age between F_1_^0ppm^ and F_1_^1ppm^ or F_1_^50ppm^ B × C females, with the exception of a significant reduction of Fe seen in liver tissue of F_1_^1ppm^ mice (Table 2).

**Table 1:** Blood concentrations of eight blood pressure-associated essential trace elements in 6-month-old B × C females.

Maternal Cd dose	Ca (mg/L)	Fe (mg/L)	K (mg/L)	Mg (mg/L)	Na (mg/L)	Cu (μg/L)	Se (μg/L)	Zn (μg/L)
**Mean blood concentrations** ± **SE**								
0 ppm	4.84 ± 0.08	49.59 ± 0.38	226.07 ± 1.8	3.41 ± 0.06	174.21 ± 1.5	59.10 ± 1.36	58.45 ± 0.97	1320.3 ± 44.9
1 ppm	4.68 ± 0.09	49.01 ± 0.68	226.53 ± 3.1	3.30 ± 0.04	174.09 ± 1.4	61.14 ± 3.18	58.17 ± 1.11	1310.7 ± 66.5
50 ppm	4.59 ± 0.14	47.73 ± 0.91	222.70 ± 4.0	3.56 ± 0.05	171.21 ± 2.8	58.79 ± 3.34	57.34 ± 1.36	1400.5 ± 49.8

**Table 2:** Liver concentrations of eight blood pressure-associated essential trace elements in 6-month-old B × C females.

Maternal Cd dose	Ca (mg/L)	Fe (mg/L)	K (mg/L)	Mg (mg/L)	Na (mg/L)	Cu (μg/L)	Se (μg/L)	Zn (μg/L)
**Mean liver concentrations** ± **SE**								
0 ppm	3.18 ± 0.06	10.83 ± 0.21	316.53 ± 3.5	22.99 ± 0.23	70.25 ± 1.3	549.2 ± 8.6	186.93 ± 2.7	3731.6 ± 65.9
1 ppm	3.13 ± 0.08	**10.00** ± 0.24*	314.00 ± 5.3	22.87 ± 0.53	68.65 ± 0.8	548.1 ± 13.5	195.22 ± 3.9	3707.2 ± 76.5
50 ppm	3.14 ± 0.05	10.29 ± 0.21	324.42 ± 1.3	23.57 ± 0.19	68.07 ± 1.7	590.2 ± 12.6	194.31 ± 4.3	3917.4 ± 74.9

*p < 0.05. One-way ANOVA with post-hoc Dunnett’s test comparing 1 ppm and 50 ppm to 0 ppm.

### Maternal Cd exposure is associated with significantly altered levels of essential trace elements at birth

Due to the absence of any overt biomarkers of hypertension in adulthood, we focused on newborn F_1_ tissues to identify molecular changes in early life associated with maternal Cd exposure that may contribute to the programming of hypertension in adulthood. In addition to the 8 elements associated with blood pressure that were quantified in 6-month-old B × C female tissue, 5 additional trace elements (phosphorus [P], sulfur [S], cobalt [Co], manganese [Mn], molybdenum [Mo]) were measured in newborn blood and liver tissue to quantify all essential trace elements in the mouse that were feasible with our spectrometry methods. Strikingly, maternal Cd exposure resulted in significant differences in levels of all elements tested (Tables 3 and 4). In pup blood, Ca, Na, Zn, Co, Cu, Mn, and Mo were significantly increased due to maternal Cd exposure, while Fe, K, Mg, P, S, and Se were significantly decreased. In pup liver, Mg, Na, and Mn were significantly increased due to maternal Cd exposure; all others were significantly decreased. Elements that were significantly increased in pup blood, but significantly decreased in pup livers due to maternal Cd exposure were Ca, Zn, Co, Cu, and Mo. Mg was the only element significantly decreased in pup blood, but significantly increased in pup livers due to maternal Cd exposure. Several elements were significantly different from the controls in only the F_1_^1ppm^ pups (blood Mn, blood Mo, liver Ca, liver K, liver P, liver Co). No Cd was detected in newborn blood or liver tissue for any of the treatment groups given our reporting limits, consistent with other studies showing that the placenta acts as a barrier to the efficient maternal-fetal transfer of Cd^12^.

**Table 3:** Blood concentrations of 13 essential trace elements and Cd in newborn F1 mice maternally-exposed to Cd.

Maternal Cd dose	Ca (mg/L)	Fe (mg/L)	K (mg/L)	Mg (mg/L)	Na (mg/L)	P (mg/L)	S (mg/L)	Zn (mg/L)	Co (μg/L)	Cu (μg/L)	Mn (μg/L)	Mo (μg/L)	Se (μg/L)	Cd (μg/L)
**Mean blood concentrations** ± **SE**														
0 ppm	5.42 ± 0.05	35.4 ± 0.5	164.6 ± 2.3	4.84 ± 0.04	187.4 ± 1.2	42.0 ± 0.6	85.8 ± 0.9	0.88 ± 0.03	0.40 ± 0.05	143.0 ± 3.6	4.74 ± 0.19	4.92 ± 0.34	30.0 ± 0.8	<1.0
1 ppm	**5.75** ± 0.07**	**30.9** ± 0.9***	169.2 ± 2.3	4.99 ± 0.06	**198.2** ± 1.7***	43.2 ± 0.9	87.7 ± 1.2	0.92 ± 0.02	**0.55** ± 0.01*	152.2 ± 2.1	**6.25** ± 0.54*	**6.03** ± 0.25*	30.2 ± 0.8	<1.0
50 ppm	**7.22** ± 0.07***	**15.1** ± 0.2***	**108.1** ± 1.4***	**4.30** ± 0.07***	**255.1** ± 1.9***	**33.3** ± 0.5***	**62.0** ± 0.8***	**1.01** ± 0.04**	**0.75** ± 0.02***	**186.7** ± 4.0***	5.99 ± 0.53	5.15 ± 0.25	**26.0** ± 0.7***	<1.0

*p < 0.05, **p < 0.01, ***p < 0.001. One-way ANOVA with post-hoc Dunnett’s test comparing 1 ppm and 50 ppm to 0 ppm.

**Table 4:** Liver concentrations of 13 essential trace elements and Cd in newborn F1 mice maternally-exposed to Cd.

Maternal Cd dose	Ca (mg/L)	Fe (mg/L)	K (mg/L)	Mg (mg/L)	Na (mg/L)	P (mg/L)	S (mg/L)	Zn (mg/L)	Co (μg/L)	Cu (μg/L)	Mn (μg/L)	Mo (μg/L)	Se (μg/L)	Cd (μg/L)
**Mean liver concentrations** ± **SE**														
0 ppm	4.10 ± 0.10	11.1 ± 0.4	354.4 ± 3.4	22.0 ± 0.2	89.1 ± 1.1	308.9 ± 5.2	192.9 ± 1.7	5.08 ± 0.13	2.16 ± 0.06	1489 ± 72	19.0 ± 1.0	23.1 ± 1.3	47.9 ± 1.1	<1.0
1 ppm	**3.78** ± 0.08*	10.4 ± 0.5	**344.4** ± 2.1*	21.4 ± 0.3	89.4 ± 1.0	**279.3** ± 4.4***	**186.4** ± 2.1*	**4.63** ± 0.11**	**1.97** ± 0.04*	**1264** ± 37*	17.2 ± 0.4	**18.4** ± 1.1**	46.1 ± 0.8	<1.0
50 ppm	4.07 ± 0.11	**2.8** ± 0.1***	349.6 ± 3.3	**22.7** ± 0.2*	**95.0** ± 1.5**	295.1 ± 4.9	**185.9** ± 2.1*	**4.42** ± 0.10***	2.03 ± 0.04	**1298** ± 52*	**25.5** ± 1.5***	**14.7** ± 0.3***	**44.0** ± 1.1*	<1.0

*p < 0.05, **p < 0.01, ***p < 0.001. One-way ANOVA with post-hoc Dunnett’s test comparing 1 ppm and 50 ppm to 0 ppm.

Levels of these 13 elements were also quantified in maternal blood and liver tissues at the time of newborn pup dissections to determine if the levels observed in F_1_ pups may be a consequence of perturbed trace element homeostasis in mothers. 50 ppm Cd significantly affected maternal blood levels of Ca, Fe, K, Mg, Na, P and S; additionally, 50 ppm Cd significantly affected maternal liver levels of Fe and Zn (Tables 5 and 6). The directional change of the levels (i.e. significantly increased or decreased due to Cd) was the same between pups and mothers, with the exception of liver Zn being decreased in the pups and increased in the mothers. Cd was undetected in maternal blood at delivery for F_0_^0ppm^ and F_0_^1ppm^ females, but was detected in the F_0_^50ppm^ females at 5.23 μg/L ± 0.99 μg/L (mean ± standard error).

**Table 5:** Blood concentrations of 13 essential trace elements and Cd in mothers at delivery.

Maternal Cd dose	Ca (mg/L)	Fe (mg/L)	K (mg/L)	Mg (mg/L)	Na (mg/L)	P (mg/L)	S (mg/L)	Zn (mg/L)	Co (μg/L)	Cu (μg/L)	Mn (μg/L)	Mo (μg/L)	Se (μg/L)	Cd (μg/L)
**Mean blood concentrations** ± **SE**														
0 ppm	5.15 ± 0.26	39.16 ± 1.40	215.59 ± 8.37	4.40 ± 0.22	179.15 ± 7.25	58.34 ± 3.50	119.95 ± 2.83	0.58 ± 0.03	1.49 ± 0.61	138.25 ± 7.19	14.13 ± 2.91	5.71 ± 0.74	47.80 ± 3.92	<1.0
1 ppm	5.64 ± 0.19	37.05 ± 1.33	207.07 ± 14.25	4.11 ± 0.27	185.01 ± 4.09	54.65 ± 3.49	117.84 ± 4.64	0.60 ± 0.04	1.20 ± 0.25	134.67 ± 10.14	11.97 ± 1.53	5.51 ± 0.88	48.00 ± 1.31	<1.0
50 ppm	**6.66** ± 0.44*	**26.73** ± 0.98***	**157.33** ± 1.53**	**3.42** ± 0.07**	**227.07** ± 2.27***	**44.21** ± 0.29**	**104.04** ± 2.39**	0.55 ± 0.06	0.73 ± 0.03	141.50 ± 2.72	8.24 ± 0.50	6.18 ± 0.76	44.42 ± 1.65	5.23 ± 0.99

*p < 0.05, **p < 0.01, ***p < 0.001. One-way ANOVA with post-hoc Dunnett’s test comparing 1 ppm and 50 ppm to 0 ppm.

**Table 6:** Liver concentrations of 13 essential trace elements and Cd in mothers at delivery.

Maternal Cd dose	Ca (mg/L)	Fe (mg/L)	K (mg/L)	Mg (mg/L)	Na (mg/L)	P (mg/L)	S (mg/L)	Zn (mg/L)	Co (μg/L)	Cu (μg/L)	Mn (μg/L)	Mo (μg/L)	Se (μg/L)	Cd (μg/L)
**Mean liver concentrations** ± **SE**														
0 ppm	4.58 ± 0.54	4.52 ± 0.33	426.77 ± 51.96	31.64 ± 4.21	85.67 ± 11.96	429.91 ± 56.67	365.19 ± 44.85	4.43 ± 0.20	7.84 ± 1.21	918.25 ± 203.21	278.25 ± 36.46	94.23 ± 21.36	145.08 ± 29.30	1.52 ±0.14
1 ppm	4.58 ± 0.54	4.86 ± 0.31	358.31 ± 14.47	25.82 ± 1.43	72.65 ± 3.22	355.60 ± 16.80	285.15 ± 29.65	4.57 ± 0.14	6.57 ± 0.26	503.67 ± 24.17	292.00 ± 58.51	72.20 ± 4.55	122.33 ± 11.61	17.16 ± 1.40
50 ppm	3.86 ± 0.03	**3.27** ± 0.25**	342.58 ± 3.95	25.51 ± 0.83	73.24 ± 1.85	344.36 ± 9.48	285.08 ± 11.83	**5.31** ± 0.26**	6.21 ± 0.14	606.80 ± 54.74	299.20 ± 14.81	63.18 ± 2.02	99.58 ± 3.54	1410.80 ± 226.44

**p < 0.01. One-way ANOVA with post-hoc Dunnett’s test comparing 1 ppm and 50 ppm to 0 ppm.

### Transcriptomic analysis of newborn B × C female hearts shows Cd-associated changes in gene expression and pathways involved in heart development and hypertension

**Figure 5:**
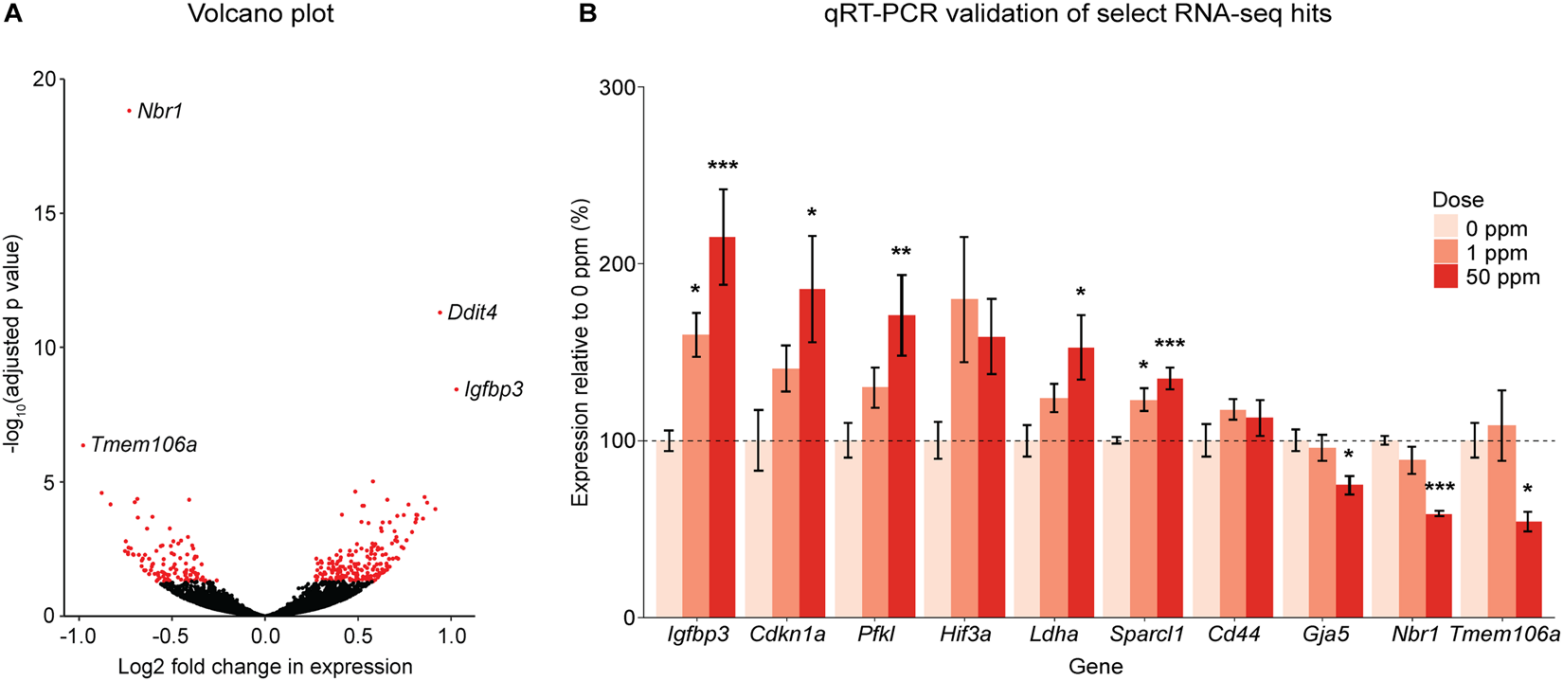
Volcano plot and qRT-PCR validation of RNA-seq data in newborn B × C female hearts. (**A**) Volcano plot of 14,698 genes identified through RNA-seq. The 302 genes identified as significantly differentially expressed (adj p val < 0.05) due to maternal Cd exposure are highlighted in red. The names of the four genes with the highest -log10(adj p val) are indicated to the right of their data points on the graph. (**B**) Select RNA-seq hits for qRT-PCR validation. *p < 0.05, **p < 0.01, ***p < 0.001 (one-way ANOVA with post-hoc Dunnett’s test comparing 1 ppm and 50 ppm to 0 ppm).

To gain a better understanding of molecular pathways that may contribute to increased heart weight at birth and susceptibility to hypertension during adulthood, RNA-seq was performed on whole hearts obtained from F_1_^0ppm^ and F_1_^50ppm^ B × C female mice at birth. Of the 14,698 genes with aligned reads passing our quality control filtering, 302 genes were found to be significantly differentially expressed between the two treatment groups (adjusted p-value < 0.05; Fig. 5A, Supplementary Table 3, Supplementary Fig. 3). Significantly more genes increased in expression (193) than decreased in expression (109) in the F_1_^50ppm^ mice compared to controls (p < 0.0001, chi-squared test, expected values to be equal). Among the differentially expressed genes, there was a significant enrichment for phenotype or disease terms related to hypertension, abnormal heart and cardiovascular system development, and nutritional disease (Supplementary Table 4). Pathways relevant to these phenotypes that had significantly enriched terms included hypoxia, altered cellular energy generation and carbon metabolism, ROS, nitric acid homeostasis, and altered metal homeostasis (Supplementary Table 4). Additional terms that were significantly enriched and relevant to published Cd-associated phenotypes included cancer^35^, cell death and survival^36,37^, connective tissue development^38^, immune response and disorders^39^, lipid metabolism^40^, organismal development and abnormalities^7^, and cell signaling^41,42^ (Supplementary Table 4). Despite undetected levels of Cd in fetal tissues, the DSigDB (Drug SIGnatures DataBase) analysis showed a significant enrichment for the term ‘Cadmium sulfate’. Cadmium sulfate is one of the highly soluble salt forms of Cd that is more readily absorbed from the intestinal lumen compared to other less soluble salt forms^43^.

Ten differentially expressed genes identified by RNA-seq with functions related to heart size and hypertension were validated by qRT-PCR in F_1_^0ppm^, F_1_^1ppm^, and F_1_^50ppm^ newborn B × C female hearts (Fig. 5B; Supplementary Table 2). All genes except two (*Hif3a*, *Cd44*) were significantly differentially expressed due to maternal Cd exposure, consistent with the RNA-seq data. Most of these showed a dose-dependent response to maternal Cd exposure, although the differences between F_1_^0ppm^ and F_1_^1ppm^ mice were significant only for two genes (*Igfbp3*, *Sparcl1*) (Fig. 5B).

## Discussion

CVD is the leading cause of death and disability worldwide^15,16^. Although there are genetic risk factors for CVD, increased CVD prevalence has been linked to environmental factors including exposure to Cd and other toxic metals^15^. Most research in humans and animal models has focused on the significance of chronic Cd exposure during adulthood. Although Cd inefficiently crosses the placenta^12^, there is growing evidence that maternal exposure to Cd can impact child development and health^2,20,41,44^. However, molecular mechanisms of Cd toxicity in this context have been challenging to study in humans.

To better understand the impact and mechanisms of action of developmental Cd exposure on cardiovascular health, we established a mouse model of maternal Cd exposure. Two doses of Cd were chosen to reflect a low and a high dose relevant to human exposure levels. Cd naturally occurs in the environment at low levels; the average amount of Cd in the Earth’s crust is estimated to be roughly 0.3 ppm^7^. However, geographic clusters can contain higher levels of Cd as shown by a study in North Carolina identifying regions with up to 1.6 ppm Cd in the soil^8^. Therefore, exposure to 1 ppm Cd can be considered a realistic level of human exposure. The mean level of blood Cd in the US population has been reported to be 0.337 μg/L^8^; this level is below our reporting limits and consistent with our inability to quantify Cd in the blood of F_0_^1ppm^ mice. The mean level of Cd detected in the blood of F_0_^50ppm^ mice was approximately 15.5 times the mean blood Cd levels of the US population; however, people living in Cd-polluted areas demonstrate blood Cd levels similar to or higher than the levels quantified here in F_0_^50ppm^ mice^45,46^. Additionally, fetal growth restriction was observed primarily in the F_1_^50ppm^ mice, a phenotype consistent with Cd exposure in humans^47^, providing further support that the high dose is relevant to the study of Cd-associated human health outcomes.

Using this model, we have shown that maternal Cd exposure is associated with increased heart weight at birth and can program hypertension during adulthood. Our data showing that maternal Cd-induced hypertension is observed only in females is consistent with sex-specific responses to Cd reported in other studies^48,49^. Hypertension was observed in maternal Cd-exposed B × C, but not C × B females; this discordance could be explained by the small sample size of F_1_^50ppm^ C × B female mice, a consequence of the negative impact of 50 ppm Cd on C female breeding performance. Alternatively, our results could reflect differences in gene-by-environment interactions between the parental inheritance of the B and C alleles, such that the maternally-inherited B allele and/or the paternally-inherited C allele is more susceptible to developmental Cd exposure. Our hybrid mouse system will provide a valuable model for dissecting the genetic basis of differences in susceptibility to Cd-induced hypertension.

To gain insight into the molecular basis of the hypertensive phenotype, we measured a marker of circulating ROS and essential trace element levels in adult female F_1_^50ppm^ B × C mice. We did not detect any differences compared to F_1_^0ppm^ controls at this age, suggesting that molecular changes in early life may be sufficient to program this phenotype. We therefore assayed trace element levels at birth and, strikingly, demonstrated perturbation of all elements measured. Many of these elements are important for cardiovascular development and function. For example, Na was significantly increased in pup blood and livers due to maternal Cd exposure; excess dietary Na is a significant contributing factor to hypertension^50^. Na concentrations also correlate with left ventricular hypertrophy^51^, which was previously seen in rats maternally-exposed to 30 ppm Cd^2^. Additionally, Se levels were significantly decreased in pup tissues in response to maternal Cd exposure; Se is a vital component of antioxidant enzymes, and diseases due to Se deficiency present with enlarged hearts, congestive heart failure, and other altered cardiac features^52^. Fe deficiency may be one of the most significant contributing factors to the reduced birth weight, increased heart weight at birth, and hypertension observed in our study. Fe deficiency is associated with increased oxidative stress that may damage cells in the heart and endothelial lining of vasculature^53^, possibly contributing to the susceptibility to hypertension. The heart responds to chronic stress by increasing in size to increase blood flow, oxygen, and nutrient delivery to tissues^54^, potentially explaining the increased heart weight at birth. Not surprisingly, a severe Fe deficiency is associated with an increased risk of CVD in humans^53^.

We propose that perturbed trace element homeostasis could represent one of the earliest events in the mechanisms leading to the phenotypes observed in this study. The direction of changes of many of the trace element levels in fetal tissues correlated with those seen in maternal tissues; this correlation highlights the importance of including a pre-conception exposure window in animal models of maternal exposure, as maternal exposures in reality often begin prior to pregnancy. Additional trace elements in F_0_^50ppm^ tissue appeared to correlate with fetal levels, but were not statistically significant, likely due to small sample sizes. Thus, many of the essential trace elements seen perturbed in the pups may be due to existing Cd-induced perturbations in the mother. It is also possible that the trace element levels are further exacerbated at the placental level, as Cd can interfere with nutrient transport across the placenta^29^.

Zn and Fe are the most well-studied elements affected by maternal Cd exposure due to their similar chemical properties to Cd and shared mechanisms of absorption^55^. One study showed that 50 ppm maternal Cd exposure in rats reduced Fe in maternal liver and whole fetuses, yet increased Zn in maternal liver^29^, consistent with the findings in our study. The study also quantified Zn and Cu in whole fetuses, but found no significant differences as a result of 50 ppm maternal Cd exposure. Here, we show a significant increase in blood Zn and Cu, yet a significant decrease in liver Zn and Cu, which could explain why no overall differences were observed in whole rat fetuses.

Our RNA-seq data from hearts at birth provide further support for early life molecular changes programming susceptibility to hypertension in adulthood. To our knowledge, this is the first time that a global transcriptomic analysis has been performed in the heart in the context of Cd exposure. We show changes in expression in genes implicated in hypertension, abnormal heart and cardiovascular system development, and increased heart size. Our RNA-seq data are further enriched for multiple mechanistic pathways that contribute to these diseases such as hypoxia, altered cellular energy, ROS, nitric acid homeostasis, and altered metal homeostasis, consistent with our trace element analysis. There was a significantly larger proportion of genes that increased in expression in response to maternal Cd exposure, consistent with a previous study in rats that found an overall increase in RNA synthesis in cardiac muscle of juvenile offspring maternally-exposed to 50 ppm Cd^56^; this is likely due to the heart needing to adapt to a new environment (e.g., hypoxia).

Mechanisms through which maternal and adult exposure to Cd affect health may differ as the placenta acts as a barrier to Cd, but no such barriers exist when exposed to Cd postnatally. Considering that little (if any) Cd enters fetal circulation, maternal Cd exposure may exert its effects indirectly, such as impacting placental development and function^57–59^. However, there was a significant enrichment for the term ‘Cadmium sulfate’ among differentially expressed genes identified by RNA-seq, indicating that maternal Cd exposure may act through similar mechanisms in the offspring as those in response to Cd exposure during adulthood.

Blood pressure was measured using the non-invasive tail cuff method to limit stress associated with surgery and recovery. The technique has been extensively used and is appropriate for measures of obtaining overall arterial pressure rather than instantaneous pressure in response to a stimulus. There are limitations to this technique; for example, tail cuff arterial pressure does not always mirror central arterial pressure^60^. Measurements are also susceptible to fluctuations in the environmental temperature, although mice were maintained on a heat pad and under a blanket during the procedure to control for this. Another limitation of our study is the small sample size of F_1_^50ppm^ C × B mice, due to the effects of Cd on C female breeding performance.

In summary, based on our assessment of trace element levels and transcriptomic analysis of newborn hearts, we propose that maternal Cd exposure leads to a nutrient-poor environment in the offspring during development, which leads to increased heart weight and susceptibility to hypertension during adulthood. Further studies using our mouse model will be required to test this hypothesis empirically. Our observations provide novel insights into mechanisms underlying Cd-associated CVD.

## Materials and Methods

### Animal husbandry, Cd exposure, and tissue collection

C57BL/6J (‘B’) and CAST/EiJ (‘C’) mice were obtained from the Jackson Laboratory and bred within their respective strain to generate the F_0_ generation. Animals were maintained on a standard 14-hour light, 10-hour dark cycle^61^ in Green Line IVC Sealsafe cage housing systems (Tecniplast) and fed LabDiet 5001 rodent diet (Granville Milling Company) ad libitum. 5- to 7-week old female F_0_ B or C mice were provided unrestricted access to filtered drinking water (Millipore RiOs Essential RO water purification system) containing 0 ppm, 1 ppm, or 50 ppm Cd in the form of CdCl_2_ (99.99% purity, Sigma-Aldrich, catalog number 202908). Multiple cohorts of females were exposed to Cd for 5 weeks. The study was not blinded, but females from all groups were treated simultaneously within their cohorts. After 5 weeks, females were mated with previously unexposed males of the opposite strain and of similar age. Cd exposure continued throughout mating and pregnancy, and was discontinued once a litter was observed (Fig. 1A). Cd exposure did not affect water consumption or female body weight during the 5-week preconception exposure window (data not shown).

Hybrid F_1_ offspring were generated to enable analyses of allele-specific gene expression and DNA methylation for a separate study (KH, MC, manuscript in preparation). The genotypes of F_1_ mice are referred to as ‘B × C’ (B mother × C father) and ‘C × B’ (C mother × B father) (Fig. 1B). F_1_ animals born to dams exposed to 0 ppm, 1 ppm or 50 ppm Cd were dissected within 24 hours of birth or at 6 months of age. Sex was determined through observation of the gonads. Tissues were weighed, flash-frozen immediately, and stored at −20 °C (for ICP-MS) or −80 °C (for all other analyses).

Sample sizes are presented in Supplementary Table 1. Animal work was approved by the North Carolina State University (NCSU) Institutional Animal Care and Use Committee under protocol 16-045-B. Experiments were conducted in accordance with the Guiding Principles in the Use of Animals in Toxicology.

### Blood pressure measurements

Blood pressures were measured on a random subset of 5.5-month-old F_1_ mice by a noninvasive tail-cuff method using a CODA 2-channel system (CODA-HT2, Kent Scientific) as described^62^. Mice were brought to the testing room and remained in their cages for 30 minutes to acclimate. Animals were restrained on a heating pad and covered with a towel to maintain body temperature. Animals were restrained for 5 minutes before measurements were taken. Blood pressure data used for analysis were taken on the fourth or fifth consecutive day of testing. Only readings in which a heart rate was detected were used. Sample sizes are presented in Supplementary Table 1.

### Serum collection, serum protein quantification, and protein carbonyl ELISA

Trunk blood of 6-month-old F_1_ mice was collected immediately after cervical dislocation and decapitation in 1.7 mL microtubes, allowed to clot for at least 15 minutes at room temperature, then centrifuged at 4 °C for 15 minutes at 2000 rcf to separate the serum. Serum was transferred to a clean microtube, then stored at −80 °C until analysis.

Serum was thawed on ice, then subjected to a BCA assay to determine protein concentration (Pierce BCA Protein Assay Kit, Thermo Scientific, 23227). Briefly, serum was diluted 1/30 in 1X autoclaved PBS (Quality Biological, A611-E404-119) to be within the working range of the BCA kit. Standards for the BCA kit were also diluted in 1X autoclaved PBS. 3 μL of the standards and serum samples were each tested in triplicate according to kit instructions. Absorbance at 562 nm was measured on a FLUOstar Omega microplate reader (BMG Labtech). Protein concentration was calculated according to kit instructions.

The diluted serum samples were further diluted in 1X autoclaved PBS to reach a final concentration of 10 μg/mL, then subjected to a protein carbonyl ELISA kit according to the manufacturer’s instructions (OxiSelect™ Protein Carbonyl ELISA Kit, Cell Biolabs, STA-310). Absorbance at 450 nm was measured on a FLUOstar Omega microplate reader (BMG Labtech). Sample sizes are presented in Supplementary Table 1.

### Quantification of elements in fetal and adult tissues

10–30 mg of blood or liver tissue collected during maternal and F_1_ dissections and then stored at −20 °C was used for this purpose. Blood was weighed after collection as measuring volume was not technically practical. All maternal and F_1_ samples were digested in high-purity nitric acid (10 μL nitric acid per 1 mg sample, Fisher Scientific, A467-500) overnight at room temperature, then submitted for analysis through the NCSU Environmental and Agricultural Testing Service Laboratory. Trace elements were determined by inductively coupled plasma mass spectrometry (ICP-MS) using a Perkin Elmer Elan DRCII, while macro elements were determined by ICP-Optical Emission Spectrometry (ICP-OES) using a Perkin Elmer ICP-OES Model 8000. Trace elements (ICP-MS) were run under standard conditions and data were normalized to non-analyte internal standards (115In or 103Rh) to correct for small differences due to instrumental drift, sample/standard solution matrices, and sample transport. A laboratory-check standard of a different stock solution was prepared and used to verify the calibration standard solution (RSD +/− 10%). A continuing laboratory check standard was analyzed every 12 samples, and the calibration curve was repeated if the value was +/− 10%. All samples were run against a 5 or 6 point multi-calibration curve with a linearity R^2^ ≥ 0.999. Macro elements run on the ICP-OES were run using a meinhardt nebulizer/cyclonic spray chamber using the same quality assurance/quality control procedure above except without internal standards. Multi-calibration standard solutions were purchased through Spex Certiprep or Inorganic Ventures, Inc. and used within the expiration date. Elements and their detection and reporting limits are listed in Table 7.

**Table 7:** Elements and their method detection and reporting limits.

Element	Method	Detection limit	Reporting limit
Cadmium (Cd)	ICP-MS	0.050 μg/L	1.0 μg/L
Cobalt (Co)	ICP-MS	0.050 μg/L	0.5 μg/L
Copper (Cu)	ICP-MS	0.080 μg/L	0.8 μg/L
Manganese (Mn)	ICP-MS	0.050 μg/L	0.5 μg/L
Molybdenum (Mo)	ICP-MS	0.1 μg/L	1.00 μg/L
Selenium (Se)	ICP-MS	0.1 μg/L	1.5 μg/L
Zinc (Zn)	ICP-MS	0.050 μg/L	1.0 μg/L
Calcium (Ca)	ICP-OES	0.020 mg/L	0.1 mg/L
Iron (Fe)	ICP-OES	0.005 mg/L	0.050 mg/L
Potassium (K)	ICP-OES	0.020 mg/L	0.2 mg/L
Magnesium (Mg)	ICP-OES	0.020 mg/L	0.1 mg/L
Sodium (Na)	ICP-OES	0.020 mg/L	0.1 mg/L
Phosphorus (P)	ICP-OES	0.050 mg/L	0.2 mg/L
Sulfer (S)	ICP-OES	0.050 mg/L	0.2 mg/L

For newborn F_1_ tissues, sex and direction of cross were determined to not significantly influence the detected levels of elements, so B × C and C × B male and female data were pooled within tissue type and treatment group. Male and female pups were equally represented in each treatment group. Maternal strain was determined to not significantly influence the levels of elements, so B and C F_0_ data were pooled within tissue type and Cd dose. Initially, only Cd, Zn, and Fe were quantified in F_0_ and F_1_ newborn tissues, but upon encouraging preliminary data, additional F_0_ B and F_1_ newborn B × C samples were tested for these three elements and all other elements described above. Sample sizes are presented in Supplementary Table 1.

### RNA extraction and quality assessment

RNA was extracted from 24 newborn B × C female whole hearts using the AllPrep DNA/RNA/miRNA kit (Qiagen, 80204). Eight 0 ppm females representing 6 litters, eight 1 ppm females representing 4 litters, and eight 50 ppm females representing 5 litters were used. Hearts stored at −80 °C were homogenized in the kit’s lysis buffer using a microtube homogenizer (Biospec 3110Bx Cell Disrupter 4800, BZ10124883). RNA was extracted from the hearts following the manufacturer’s protocol for 10–30 mg of starting material. RNA was suspended in nuclease-free water and quantified using a Nanodrop 2000. RNA purity and size integrity were determined at the NCSU Genomic Sciences Laboratory (GSL) using an Agilent 2100 Bioanalyzer with an RNA 6000 Nano Chip (Agilent Technologies). All RNA samples had an RNA Integrity Number (RIN) ≥ 9.9.

### RNA-seq

Total RNA from hearts of four F_1_^0ppm^ and four F_1_^50ppm^ B × C female mice (each individual representing a different litter) was submitted to the NCSU GSL for indexed library construction and sequencing. Purification of messenger RNA (mRNA) was performed using oligo-dT beads provided in the NEBNext Poly(A) mRNA Magnetic Isolation Module (New England Biolabs [NEB]). Complementary DNA (cDNA) libraries for Illumina sequencing were constructed using the NEBNext Ultra Directional RNA Library Prep Kit (NEB) and NEBNext Multiplex Oligos for Illumina (NEB) using the manufacturer-specified protocol. Following adapter ligation, the samples were selected for a final library size (adapters included) of 400–550 bp using sequential AMPure XP bead isolation (Beckman Coulter). Library enrichment was performed and indexes for each sample were added during the protocol-specified PCR amplification. Amplified library fragments were purified and checked for quality and final concentration using an Agilent 2200 Tapestation (D1000 chip, Agilent Technologies) combined with a Qubit fluorometer (ThermoFisher). Libraries were pooled in equimolar amounts for clustering and sequencing on an Illumina HiSeq. 2500 DNA sequencer, utilizing a 125 bp single-end cycle sequencing kit (Illumina). The software package Real Time Analysis (RTA) was used to generate raw bcl, or base call files, which were then de-multiplexed by sample into fastq files using bcl2fastq Conversion Software v2.17 (Illumina).

Raw sequencing reads were transferred to the Cyverse Discovery Environment (DE) using Cyberduck. FastQC was performed using the FastQC 0.11.5 (multi-file) application (app). The Trimmomatic-programmable-0.33 app was used to trim the reads using the parameters HEADCROP:10, SLIDINGWINDOW:4:20, and MINLEN:20. FastQC was performed again to verify the quality of trimmed reads. The Mouse RNASeq DeSEQ2 pipeline published by Cyverse^63^ was used for indexing, aligning, read counts, and differential expression analysis; all analyses were performed using the recommended parameters. The HISAT2-index-align-2.1 app was used to index and align reads to the mouse reference genome (GRCm38.6 assembly). Aligned reads were counted using the HTSeq-count-0.6.1 app. Differential gene expression analysis was performed using the DeSEQ2 (multifactorial pairwise comparisons) app. A statistical report of this analysis is presented in Supplementary File 1 and the results of the DeSEQ2 analysis are presented in Supplementary Table 3.

The 302 genes that were significantly differentially expressed (adjusted p value < 0.05) between 0 ppm and 50 ppm maternal Cd exposure groups were used as input for enrichment analysis using Ingenuity Pathway Analysis (IPA; Qiagen) and Enrichr^64,65^. The KEGG, GO biological process, GO molecular function, GO cellular component, DSigDB, and MGI mammalian phenotype database results were used from the Enrichr analysis. Only enrichments with significant adjusted p values (<0.05) were considered. 10 genes that frequently occurred across multiple relevant terms and categories were chosen for qRT-PCR validation. Supplementary Fig. 3 was generated using the 302 significantly differentially expressed genes using http://www.heatmapper.ca/ using average linkage clustering method and Pearson distance measurement method.

### qRT-PCR

100 ng of total RNA extracted as described above from 24 B × C female newborn pup hearts was used as a template to synthesize first strand cDNA. DNase I treatment was performed prior to reverse transcription as part of the Qiagen AllPrep kit protocol. Random primers (Promega, C118A) were annealed to the DNase I-treated RNA, then the RT reaction was performed according to the manufacturer’s protocol (M-MLV RT enzyme, Promega, M170B). qRT-PCR was performed on a Real-Time 7300 machine (Applied Biosystems) using SsoAdvanced Universal SYBR Green Supermix (Bio-Rad, 1725271). The 20 μL RT-qPCR reaction mix per well was prepared according to the manufacturer’s protocol for SYBR Green. 1 μL of the cDNA for the standards or the tested samples was included in the 20 μL reaction. cDNA of the tested samples was diluted 1/10, while the standards were diluted 1/5, 1/10, 1/20, 1/40, and 1/80. Standards, the no template control (NTC), and all other tested samples were each run in triplicate. The cycling conditions were as follows: 95 °C for 30 seconds; 40 cycles of 95 °C for 15 seconds, 60 °C for 30 seconds; dissociation curve of 60.0 °C–95.0 °C. The primer sequences are provided in Supplementary Table 5. The dissociation curve confirmed that the primers amplified a single PCR product, or no product in the NTC. Amplification efficiencies were calculated by determining the slope of the regression between the log values of the standard concentrations and the average Ct value of the standards. *Polr2a* was not significantly differentially expressed between treatment groups (data not shown) and was used as a reference gene. Quantification of expression was calculated using the ΔΔCt method^66^.

This study was performed in compliance with MIQE standards^67^ (see Supplementary Table 6).

### Statistical analysis

All statistical analyses were performed using a one-way analysis of variance (ANOVA) followed by Dunnett’s multiple comparison test using R software, comparing animals from the 1 ppm and 50 ppm groups to 0 ppm controls. Outliers, as defined by a point which falls more than 1.5 times the interquartile range above the third quartile or below the first quartile, were omitted from the analysis. B × C litters under n = 6 and C × B litters under n = 3 were omitted from raw or proportional weight analyses. Data are presented as the mean ± standard error of the mean. Modeling the data using JMP software (SAS Institute Inc.) showed no significant effects of litter on the measured endpoints.

## Supplementary information

Supplementary File 1

Supplementary Figures

Supplementary Table 1

Supplementary Table 2

Supplementary Table 3

Supplementary Table 4

Supplementary Table 5

Supplementary Table 6
