# Supplementary material for: Maternal cadmium exposure in the mouse leads to increased heart weight at birth and programs susceptibility to hypertension in adulthood
Source: Sci Rep. 2019 Sep 19;9:13553. doi: 10.1038/s41598-019-49807-5 (PMC6753073; doi:10.1038/s41598-019-49807-5)
Supplement: Supplementary file 2 — Supplementary Figures [file 41598_2019_49807_MOESM2_ESM.pdf]

Supplementary Figures

Maternal cadmium exposure in the mouse leads to increased heart weight at birth and programs susceptibility to hypertension in adulthood

Kathleen M. Hudson[1,2], Scott M. Belcher[1], Michael Cowley[1,2*]

[1]Center for Human Health and the Environment, and Department of Biological Sciences, North Carolina State University, Raleigh, NC, 27695, USA.
[2]W. M. Keck Center for Behavioral Biology, North Carolina State University, Raleigh, NC, 27695, USA.

Supplementary Figure 1. A, Litter size in each treatment group. Each dot represents a different litter. Mean litter sizes for each treatment group are indicated with red lines. **B,** The effect of Cd dose on sex ratio among F_1 mice.

Supplementary Figure 2. Raw heart weight at birth and 6 months of age following maternal Cd exposure. **A,** Heart weight at birth. **B,** Heart weight at 6 months of age. *p<0.05, **p<0.01, ***p<0.001 (one-way ANOVA with post-hoc Dunnett's test comparing 1 ppm and 50 ppm to 0 ppm).

Supplementary Figure 3. Heatmap of 302 significantly differentially expressed genes in newborn BxC female whole hearts as a result of 50 ppm maternal Cd exposure.

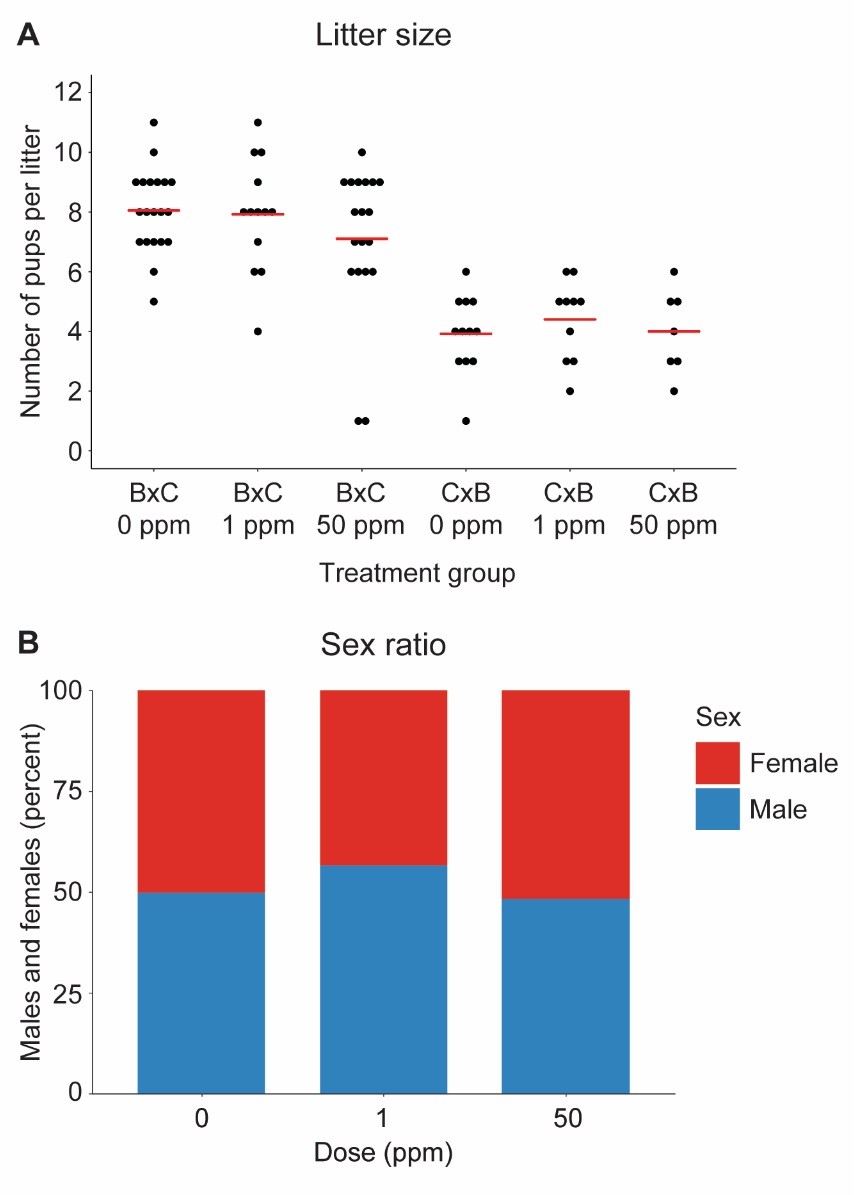

Supplementary Figure 1

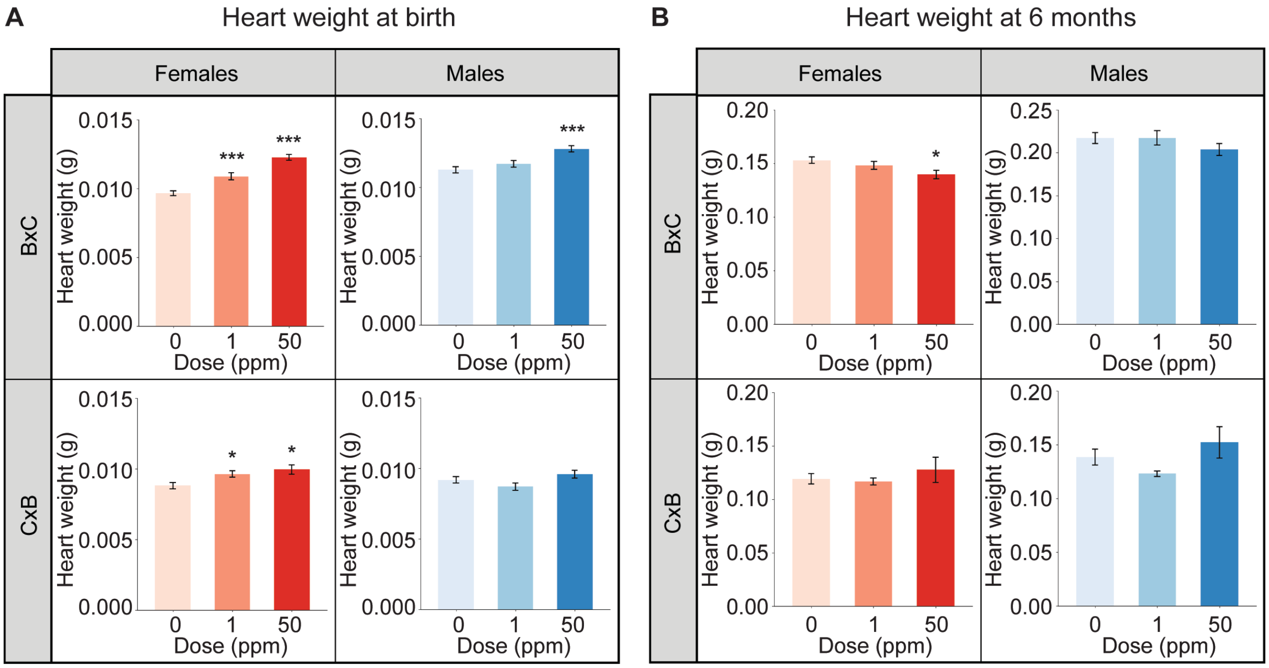

Supplementary Figure 2

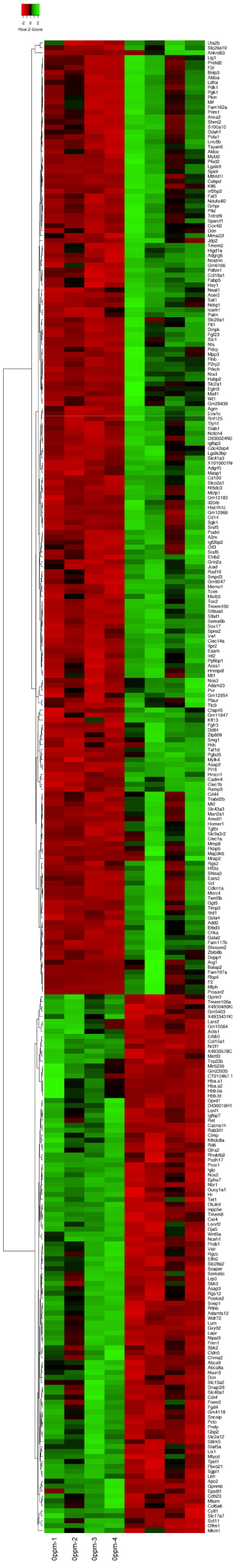

Supplementary Figure 3
